# A Combination of *Rosa Canina* Extracts and Gold Complex Favors Apoptosis of Caco-2 Cells by Increasing Oxidative Stress and Mitochondrial Dysfunction

**DOI:** 10.3390/antiox9010017

**Published:** 2019-12-24

**Authors:** Inés Mármol, Nerea Jiménez-Moreno, Carmen Ancín-Azpilicueta, Jesús Osada, Elena Cerrada, María Jesús Rodríguez-Yoldi

**Affiliations:** 1 Department of Pharmacology and Physiology, Veterinary Faculty, University of Zaragoza, 50009 Zaragoza, Spain; ines.marmol9@gmail.com; 2 Department of Science, Public University of Navarra, INAMAT, 31006 Pamplona, Spain; nerea.jimenez@unavarra.es (N.J.-M.); ancin@unavarra.es (C.A.-A.); 3 Department of Biochemistry and Molecular Biology, Veterinary Faculty, University of Zaragoza, 50009 Zaragoza, Spain; josada@unizar.es; 4 Centro de Investigación Biomédica en Red - Obesidad y Nutrición (CIBERobn) (ISCIII), IIS Aragón, IA2, 50009 Zaragoza, Spain; 5 Department of Inorganic Chemistry, Sciences Faculty, University of Zaragoza, 50009 Zaragoza, Spain

**Keywords:** apoptosis, autophagy, colorectal cancer, rosehip, gold complex, ROS

## Abstract

Given the alarming increase in colorectal cancer (CRC) worldwide, novel therapies are urgently needed. Plant-derived extracts have gained considerable interest in the last years due to their strong anticancer effect mediated by their unique bioactive compounds. Specifically, rosehips from *Rosa canina* have been successfully tested against several cancer models, including colon cancer. Moreover, gold derivatives are a promising alternative to the current platinum-based drugs commonly used in CRC chemotherapy due to their lack of affinity for DNA. Herein we have investigated the antitumor potential of a drug combination made of acidic polyphenols extracted from *R. canina* and the gold complex (Au(C≡C-2-NC_5_H_4_) (PTA)) in Caco-2 cell line as a model of CRC. The combination triggered strong apoptosis mediated by a blockage of the autophagic flux, which might be a consequence of a reactive oxygen species (ROS) increase and mitochondrial dysfunctionality. Our results suggest that the clinical application of plant polyphenols might enhance the anticancer effect of metallodrugs and reduce drug exposure time and therefore its side effects.

## 1. Introduction

The high incidence of cancer worldwide, along with the incomplete efficacy of current therapies, drives the development of novel clinical strategies to reduce mortality and improve patients’ quality of life. A growing body of evidence supports the potential use of certain phytocompounds that are able to kill cancer cells by inhibiting key enzymes or altering their genomic expression [1,2]. Therefore, the use of whole-plant extracts and/or isolated fractions that contain more than one bioactive compound might be a better approach to cancer therapy due to the additive effect of single agents. In line with this thinking, plant extracts have been proposed as promising adjuvants for chemotherapeutic drugs in order to enhance their effectiveness [3,4] as well as to improve its cytotoxicity on healthy tissue [5,6].

*Rosa canina* is a widespread wild plant whose rosehips have been used in traditional medicinal remedies in Europe, Asia, and North America [7]. Rosehips have a high content of ascorbic acid, phenolic compounds and other bioactive molecules that are considered responsible for their therapeutic potential. In the last years, rosehips from *R. canina* have been successfully tested on various cancer cell lines including colon, lung and prostate, suggesting their potential role on chemotherapy [8]. 

Among all types of tumor, colorectal cancer (CRC) is the second most diagnosed cancer in women and the third in men, and is the fourth most common cause of all cancer-related deaths. Although a small percentage of all diagnosed cases are due to inherited mutations, in most cases CRC onset is closely related to lifestyle factors including consumption of red meat, sedentarism or tobacco smoking. In light of this, the fast increase of CRC in developing countries due to the acquisition of so-called ‘Western-like’ lifestyle habits is not surprising but is a matter of concern [9,10,11]. CRC therapy usually consists of a combination of surgery and chemotherapy, with the cisplatin analogue oxaliplatin being one of the most common drugs. However, the use of platinum-containing drugs is associated with severe side effects due to their lack of selectivity for tumor cells. Gold-based drugs have been suggested as an alternative, since instead of interacting with DNA these complexes display high affinity for proteins overexpressed on tumor cells such as thioredoxin reductase or the proteasome system [12,13]. Therefore, gold derivatives might be considered as a safer therapeutic strategy than the current platinum-containing drugs. 

Given the promising antitumoral potential of combinations of metallodrugs and plant extracts [3,4,14] along with our previous work, we have developed a new chemotherapy combination focused on CRC treatment. This drug combination is composed of the alkynyl gold(I) complex (Au(C≡C-2-NC_5_H_4_)(PTA)) (PTA = 1,3,5-triaza-7-phosphadamantane) [15,16] and acidic polyphenols (AP) extracted from the rosehips of *Rosa canina* [17]. The objectives of the present work were the analysis of the combined effect of the acidic polyphenols and the gold complex in order to assess the AP fraction as a potential adjuvant agent for organometallic-based chemotherapy, the characterization of the effect of the resulting drug combination in terms of the type of cell death, and the putative mechanisms of action. This study has been carried out in the human colorectal adenocarcinoma cell model Caco-2 cell line. 

## 2. Materials and Methods 

### 2.1. Preparation, Purification and Fractioning of R. canina Acidic Polyphenols

The work was carried out with rosehips from the plant species *Rosa canina* harvested in the year 2014. The company, which provided the rosehip samples, is Herbes del Molí (Alicante, Spain) and the company has the necessary permissions for this type of activity. The samples do not belong to a species in danger of extinction nor are they protected. The rosehips were collected in the mountain of Mariola in a landscape called "Ca’l retor", located in the municipality of Agres (Alicante), with the permission of the owner, D. Fidel Pascual Molins. In that geographical zone, the winters are cold, with temperatures as low as −15 °C, while the summers are hot, with temperatures that can go higher than 35 °C or even 40 °C. Rainfall is irregular and can vary from 350 to 900 mm/annually. Freshly collected rosehips were stored at −40 °C prior to analysis.

The composition of polyphenols from the rosehip sample of *Rosa canina* used in the present study is shown by Jiménez et al. [17]. The average values in μg/kg dried fruit of the polyphenols are: myricetin, 5.4; rutin 22; catechin 11.9; quercetin 1.5; vanillic acid 260; caffeic acid 2; syringic acid 110; gallic acid 298; ellagic acid 80 and protocatechuic acid 210. To carry out this study only the acidic fraction of the polyphenols present in the rosehips was taken (vanillic acid, caffeic acid, syringic acid, gallic acid, ellagic acid and protocatechuic acid), which was separated from the rest of the neutral polyphenols. The separation of both fractions is described in Jiménez et al. [17].

### 2.2. HPLC Analysis of Polyphenols of Rosehips

Analysis was performed using high-pressure liquid chromatography (Waters Chromatography Div., Milford, MA, USA) equipped with two 515 pumps, a 717 autosampler and a 996 Photodiode Array Detector. Two mobile phases A (0.1% phosphoric acid) and B (acetonitrile) both from Scharlab, were used. The flow rate was 1 mL/min with the linear gradient profile: 0–20 min, from 10% to 22% B; 21–40 min, from 22% to 40% B; 41–50 min, from 40% to 55% B; 51–60 min, from 55% to 10% B; 61–65 min, equilibration at 10% B. The injection volume of sample, dissolved in methanol, was 10 μL. The column used was a reverse phase Atlantis dC18 (150 mm × 4.6 mm i.d., 5 μm particle size). The chromatographic control was performed with Empower 2.0 software (Waters, Milford, MA, USA). The criteria for identification of the different compounds was the double coincidence of the UV-visible spectrum at wavelengths characteristic of each compound and the chromatographic retention times compared with corresponding standards. The quantification was achieved using calibration curves at five different concentrations of the standard.

### 2.3. Synthesis of (Au(C≡C-2-NC_5_H_4_)(PTA))

The gold complex was prepared as previously described [15]. Briefly: to a solution of KOH (0.4 mmol) in MeOH (10 mL) containing 2-ethynylpyridine (0.3 mmol) was added [AuCl(PTA)] (0.26 mmol). After stirring the mixture for ~20 h the solid was isolated by filtration and washed with diethyl ether.

### 2.4. Cell Culture

Human Caco-2 cell line (TC7 clone) was kindly provided by Edith Brot-Laroche (Université Pierre et Marie Curie-Paris 6, UMR S 872, Les Cordeliers, France). Caco-2 cells were maintained in a humidified atmosphere of 5% CO_2_ at 37 °C. Cells (passages 50–80) were grown in Dulbecco’s Modified Eagles Medium (DMEM) (Gibco Invitrogen, Paisley, UK) supplemented with 20% fetal bovine serum (FBS), 1% non-essential amino acids, 1% penicillin (1000 U/mL), 1% streptomycin (1000 μg/mL) and 1% amphotericin (250 U/mL). The cells were passaged enzymatically with 0.25% trypsin-1 mM EDTA and sub-cultured on 25 cm^2^ plastic flasks at a density of 2 × 10^4^ cells/cm^2^. Culture medium was replaced every 2 days. Drug treatments were added 24 h post-seeding for assays on undifferentiated cells; for assays on differentiated cells, treatments were added 1-week post-seeding on 96-wells plates [18]. Cell confluence (80%) was confirmed by optical microscopy observance (Figure S1). 

### 2.5. Cell Treatment

Acidic polyphenols from *R. canina* were diluted in cell culture medium to the required concentration (IC_50_ value: 125 μg/mL) [17]. 

Gold complex was diluted in dimethyl sulfoxide (DMSO) at 2 mM and then in cell culture medium to the required concentration (IC_50_ value: 3.8 μM) [16]. 

For treatment with the drug combination, the required concentrations of acidic polyphenols and gold complex were added to the same falcon tube with cell culture medium, then vortex and incubated with Caco-2 cells. For isobologram plotting [19,20], IC_50_ of GC was re-calculated in presence of two concentrations of AP, namely 62.5 and 31.25 μg/mL. The obtained values were 1.26 and 2.04 respectively. Then, the additivity line was plotted using the original IC_50_ values for both GC and AP and the location of the points (1.26, 62.5) and (2.04, 31.25) suggested an additive effect. 

### 2.6. Cell Viability Assay

For cytotoxicity screening assays, Caco-2 cells were seeded in 96-well plates at a density of 4 × 10^3^ cells/well. Culture medium was replaced with medium containing drug panel, and cells were incubated for 24, 48 or 72 h. Antiproliferative effect was measured with the sulforhodamine B (SRB) assay as previously described [17]. Absorbance at 540/620 nm was measured with SPECTROstar Nano (BMG Labtech, Ortenberg, Germany). The effect on cell growth was expressed as a percentage of the control.

### 2.7. Measurements of Apoptosis

Caco-2 cells were seeded in 25 cm^2^ flasks and then exposed to drug panel for 48 h, then collected and stained with annexin V-FITC and propidium iodide according to manufacturer’s instruction. Cells were then transferred to flow cytometry tubes and washed twice with phosphate saline buffer (PBS), followed by a resuspension in 100 µL of annexing V-binding buffer (100 mM HEPES/NaOH pH 7.4, 140 nM NaCl, 2.5 mM CaCl_2_). Additions of 5 µL annexin V-FITC and 5 µL propidium iodide were made to each tube. After 15 min of incubation at room temperature covered from light, 400 µL of annexin-binding buffer were added to each sample and signal intensity was analyzed within 1 h with FACSARIA BD. Data were analyzed with FACSDIVA BD (v7, BD Franklin Lakes, NJ, USA).

### 2.8. Analysis of the Activation of Caspase 3

Caco-2 cells were seeded in 25 cm^2^ flasks and then exposed to drug panel for 48 h, then collected and fixed with 0.01% formaldehyde for 15 min. After 5 min centrifugation at 300 g, the pellet was resuspended in 100 µL of 0.5% v/v digitonin solution and incubated 15 min protected from light. Cells were washed with 0.1% v/v digitonin solution and centrifuged for 5 min at 300 g. The pellet was resuspended in 0.1% digitonin and anti-caspase 3 (Novus, clone C92-605) was added to each sample and incubated for 1h. Cells were then centrifuged 5 min at 500 g, washed twice with PBS and resuspended in PBS. Fluorescence was analyzed with FACSARRAY BD equipped with an argon ion laser. Ex: 394–400; Em: 495–505.

### 2.9. Cell Cycle Analysis

Caco-2 cells were seeded in 25 cm^2^ flasks and then exposed to the drug panel for 48 h, then collected and fixed in 70% ice-cold ethanol at 4 °C for 24 h. After 5 min centrifugation at 2500 rpm, cells were rehydrated in PBS and stained with 50 µg/mL propidium iodide solution. Red fluorescence was collected with a 620 nm long pass filter as a measure of the amount of PI bounded to DNA. Percentage of cells on each cell cycle phase was determined with MODIFIT 3.0 verity software (BD Franklin Lakes, NJ, USA). 

### 2.10. Determination of RIP-1 Protein Amount by Flow Cytometry 

Caco-2 cells were seeded in 25 cm^2^ flasks and then exposed to the drug panel for 48 h, then collected and the determination of receptor–interaction protein kinase 1 (RIP-1) levels was performed as previously described [16].

### 2.11. Measurement of Autophagosome Formation

Caco-2 cells were seeded in 96-well plates at a density of 4 × 10^3^ cells/well and then incubated with the drug panel for 1 or 24 h. For autophagosomes formation, Autophagy Assay Kit (Sigma–Aldrich, San Luis, MO, USA) was used according to manufacturer’s instruction. Fluorescence intensity (λ_ex_ = 360/λ_em_ = 520 nm) was measured with FLUOstar Omega (BMG Labtech, Ortenberg, Germany). Fluorescence intensity was normalized with cell viability for assays at 24 h incubation. The obtained values of fluorescence intensity are considered a reflection of total autophagosome formation.

### 2.12. Determination of Intracellular Levels of Reactive Oxygen Species (ROS)

Caco-2 cells were seeded in 96-well plates at a density of 4 × 10^3^ cells/well and then incubated with drug panel for 1 or 24 h. Determination of intracellular H_2_O_2_ formation was performed using the dichlorofluorescein (DCF) assay as previously described [21]. Fluorescence intensity (λ_ex_ = 485/λ_em_ = 535 nm) was measured with FLUOstar Omega (BMG Labtech, Ortenberg, Germany), and was normalized with cell viability for assays at 24 h incubation. The obtained values of fluorescence intensity are considered a reflection of total intracellular reactive oxygen species (ROS) content. 

### 2.13. Flow Cytometry Mitochondrial Membrane Potential Assay 

Caco-2 cells were seeded in 25 cm^2^ flasks and then exposed to drug panel for 24 or 48 h, then collected and washed twice with PBS. The pellet was resuspended in PBS at a concentration of 10^6^ cell/mL and 5 μL of 10 μM 1,1’,3,3,3’-hexamethylindodicarbo-cyanine iodide (DiIC1) were added to each sample. Tubes were incubated at 37 °C for 15 min and 400 μL PBS were added prior to analyze fluorescence with FACSARRAY BD equipped with an argon ion laser, Ex: 633 nm; Em: 658 nm. 

### 2.14. Determination of Lysosome Alkalization 

Caco-2 cells were seeded in 25 cm^2^ flasks and then exposed to the drug panel for 24 or 48 h, then collected and changes in lysosomal acid content measured with LysoTracker Green DND-26 (Molecular Probes™ L7526). The dye was first diluted on DMEM to a concentration of 75 nM. Then, 1 mL of diluted LysoTracker was added to each 50 μL of cell sample. Cells were incubated 2 h at room temperature kept in darkness and then were centrifuged 5 min at 1200 rpm. Pellet was resuspended on 200 μL phosphate buffered saline (PBS) and fluorescence signal (λ_ex_ = 504/λ_em_ = 511 nm) was analyzed by FACSARIA BD equipped with an argon ion laser.

### 2.15. Statistical Analysis

All assays were performed at least three times. Data are presented as mean ± SD. Data were subjected to one-way analysis of variance (ANOVA) and LSD–Fisher post hoc test. Differences were considered significant at *p* ≤ 0.05.

## 3. Results and Discussion

### 3.1. Acidic Polyphenols from R. canina Enhance the Antiproliferative Effect of Gold Complex

The antiproliferative effect of the acidic polyphenols fraction (AP) of rosehips from *R. canina* on Caco-2 cell line after 72 h of incubation were previously evaluated by us [17], reading an IC_50_ value of 125 μg/mL. On the other hand, the effect of the gold complex (Au(C≡C-2-NC_5_H_4_)(PTA)) towards the same cell model displayed a higher in vitro effect than plant extracts with an IC_50_ value of 3.8 μM [16]. 

**Figure 1:**
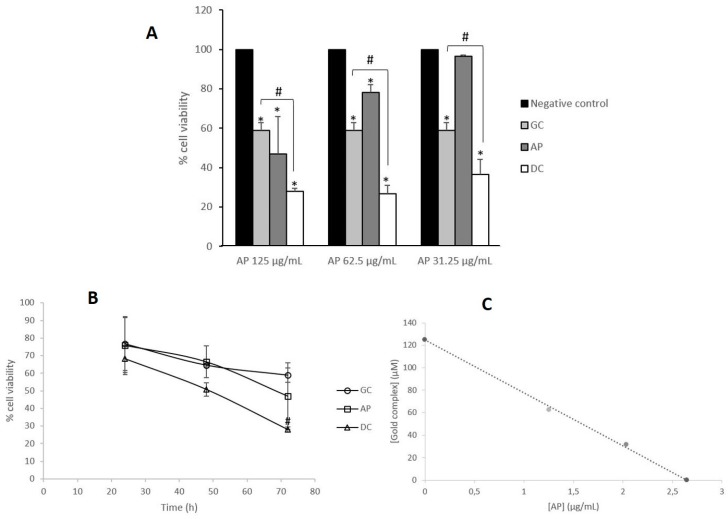
Analysis of the antiproliferative effect of the combination of gold complex and *Rosa canina* extracts. (**A**) Measurement of changes in cell viability of Caco-2 cells after 72 h incubation with 3.8 μM of gold complex (GC) and a range of concentrations (125, 62.5 and 31.25 μg/mL) of acidic polyphenols (AP). * *p* < 0.05 vs negative control. # *p* < 0.05 vs gold complex (GC). (**B**) Isobologram analysis of the cytotoxicity of the combination of gold complex and acidic polyphenols (DC). (**C**) Time-course (24, 48 and 72 h) determination of changes in Caco-2 cells viability upon treatment with 3.8 μM of the gold complex (GC), 125 μg/mL of acidic polyphenols (AP) and a combination of both (DC) at the indicated concentrations. # *p* < 0.05 vs gold complex (GC).

Initially, a range of concentrations of acidic polyphenols (125, 62.5 and 31.25 μg/mL) was tested in combination with the gold complex at its IC_50_ value for 72 h (Figure 1A). Results obtained showed the key role of AP concentration on the antiproliferative effect of drug combination, since all the tested concentration values decreased cell viability in comparison to both negative control and cells treated with the gold complex as a single agent (*p* < 0.05). In order to analyze whether the observed results were a consequence of synergy or additivity, the kind of pharmacological interaction between single agents was further studied with an isobologram (Figure 1B), after re-calculation of the corresponding IC_50_ values of the gold complex in the presence of both concentrations of AP (62.5 and 31.25 μg/mL), which afforded the IC_50_ values of 1.25 and 2.04 μM respectively, versus the previous value of 3.8 μM obtained with the gold complex alone. As can be observed on Figure 1B, the combination of the gold complex and AP showed additive cytotoxicity on Caco-2 cell line.

Then, time-course analyses were performed after 24, 48 and 72 h of incubation (Figure 1C). Incubation time was found to be crucial for the antiproliferative effect of the drug combination, since it induced significant changes in cell viability (*p* < 0.05) at short-time incubation (24 h) whereas for the gold complex these changes were not observed until 48 h. Moreover, the drug combination induced a 50% reduction on cell viability upon 48 h of incubation, whereas single agents reached that effect after 72 h. 

Caco-2 cells undergo spontaneous differentiation after reaching confluence and acquire the morphology and enzymatic profile of a mature enterocyte [22]. Thus, differentiated Caco-2 cells are considered as an acceptable model of the intestinal barrier and have been traditionally used to evaluate the toxicity of novel drugs on a non-cancerous model [23]. The drug combination and AP did not induce significant changes on cell proliferation of differentiated Caco-2 cells after long-term incubation (72 h) (Figure S2), which might suggest that such combination displays selectivity for this cancer type. It has been reported that plant extracts and plant metabolites might display a different behavior on cancer and non-cancer cells. As a part of normal diet, they are well-tolerated by non-cancer cells, whereas are harmful for cancer cells. Ivanova et al. [24] observed resveratrol-induced cell death of Jurkat cells but not influenced cell viability rates of normal lymphocytes. In this context, plant extracts have been proposed as co-adjuvants to ameliorate side effects caused by chemotherapeutic drugs such as cisplatin given their antioxidant and anti-inflammatory properties [25,26]. In the specific case of differentiated Caco-2 cells, Di Nunzio et al. [27] found that olive pomace successfully protected this intestinal barrier model from inflammation induced by the addition of interleukin-1β. Therefore, it is feasible to suppose that *R. canina* extract might protect differentiated Caco-2 cells by the oxidative and/or inflammatory damage induced by the gold complex.

### 3.2. Drug Combination Triggers Apoptosis on Caco-2 Cells

We found it interesting to analyze the type of cell death induced by the combination of the extracts and the gold complex on undifferentiated Caco-2 cells.

**Figure 2:**
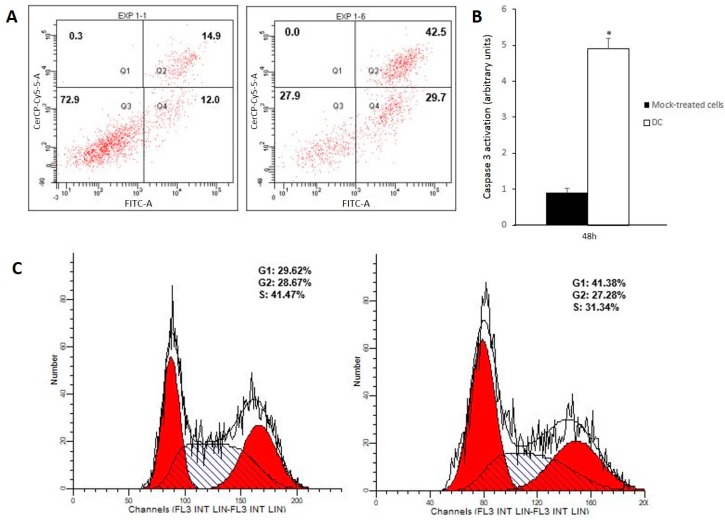
Cell death studies. (**A**) Flow cytometry histogram corresponding to Caco-2 cells stained with annexin V/propidium iodide 48 h after incubation with DMSO (negative control, left) and drug combination (DC, right). X-axis annexin-FITC and Y-axis propidium iodide. Q1: necrotic cells. Q2: late apoptotic cells. Q3: living cells. Q4: early apoptotic cells. Percentages of cell population in each condition are included. (**B**) Analysis of caspase 3 activation of Caco-2 cells 48 h after incubation with DC; * *p* < 0.05 vs negative control. (**C**) Flow cytometry cell cycle analysis with propidium iodide staining of Caco-2 cells 48 h after incubation with DC (control, left). Percentages of cell population in each cell cycle phase are included.

After 48-h incubation with drug combination, 2.8-fold increase in late apoptotic population and a 2.5-fold increase in early apoptotic cells were observed, whereas no significant changes in the necrotic cell population were found (Figure 2A). Furthermore, a 5.44-fold increase in active caspase 3 (Figure 2B) along with cell cycle arrest on G_1_ phase (Figure 2C) were observed at 48 h. Taken together, these results suggest that the drug combination induced apoptosis on Caco-2 cells. Its pro-apoptotic effect might be a direct consequence of the concomitant administration of the gold complex and the acidic polyphenols fraction, since incubation with single agents did not induce significant changes on apoptotic cells population after 48 h of incubation (Figure S3).

In a previous work [16], we reported that 24 h incubation with the gold complex (Au(C≡C-2-NC_5_H_4_)(PTA)) induced Caco-2 cell death by a programmed form of necrosis called necroptosis, which is caspase-independent. Interestingly, treatment of cell culture with the drug combination for 48 h did not modify the number of cells with activated receptor–interaction protein kinase 1 (RIP-1), one of the key regulators of necroptosis [28], which suggests that the necroptotic pathway is non-activated by the drug combination (Figure S4). Moreover, pre-incubation of cell culture with the RIP-1 inhibitor necrostatin-1 (Nec-1) (50 μM, 1h) resulted in no significant changes in cell viability in comparison with the administration of drug combination in the absence of Nec-1 (Table 1), which further confirmed that no necroptosis was triggered by the drug combination. 

**Figure 3:**
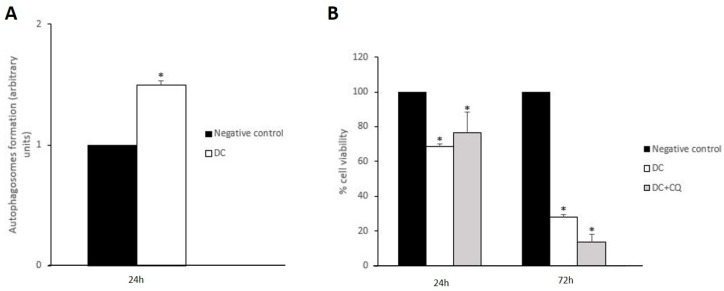
Analysis of autophagy induction. (**A**) Measurement of changes in the formation of autophagosomes on Caco-2 cells 24 h after incubation with DC. * *p* < 0.05 vs negative control. (**B**) Measurement of cell viability of Caco-2 cells upon 24 h and 72 h treatment with drug combination (DC) in presence/absence of chloroquine (10 μM, 1 h pre-incubation); * *p* < 0.05 vs negative control. No significant changes were observed in comparison to cells DC with/without CQ.

Shen and Codogno [29] found that the inhibition of necroptosis led to cell death by apoptosis. In light of this finding, the high rates of apoptotic cells observed on Figure 2A might be a consequence of the disruption of the necroptotic process induced by the concomitant administration of acidic polyphenols and the gold complex. In line with this, autophagy seems to play a key role in both inhibition and promotion of other forms of cell death given its dual cytoprotective and cytotoxic nature. Some authors have reported that activation of cytoprotective autophagy might be related to necroptosis inhibition [30,31]. Since 48 h of treatment with the drug combination resulted in no evidence of necroptosis activation, a likely activation of cytoprotective autophagy must be an upstream event. Therefore, changes in autophagosome formation were analyzed after 24 h incubation. As can be observed in Figure 3A, the drug combination induced a 1.5-fold increase in autophagosomes formation, whereas the gold complex and AP separately did not induce any significant changes (Figure S5). Consequently, the concomitant administration of both drugs triggers autophagy, which might be related to the absence of necroptosis previously discussed. 

Interestingly, pre-incubation of Caco-2 cells with the autophagy inhibitor chloroquine (CQ) (10 μM, 1 h) resulted in no significant changes in cell viability when comparing with cells non-treated with CQ after 24 h or 72 h treatment with the drug combination (Figure 3B). The autophagic process consists of two stages: the early stage comprises the formation of the autophagosome, which engulfs damaged subcellular structures as well as protein aggregates, whereas in the late stage the fusion of autophagosome and lysosome ends with the digestion of the non-functional cellular components [32]. Given that CQ inhibits late autophagy by preventing the autophagosome–lysosome fusion, our data suggest that the autophagic process is initiated upon treatment with the drug combination, although autophagy might not display a significant role on its antiproliferative effect. In fact, uncompleted autophagy has been related to high rates of apoptosis due to the accumulation of non-functional cellular structures [33,34]. Therefore, it is feasible to hypothesize that the observed strong pro-apoptotic effect induced by the drug combination (Figure 2A) might be a consequence of a blockage of the autophagic flux. 

### 3.3. Drug Combination Disrupts Redox Homeostasis, Which Induces Mitochondrial Disturbances along with Lysosomal Dysfunction

In an effort to determine whether treatment with the drug combination triggered uncompleted autophagy, we analyzed lysosomal integrity 24 h and 48 h after incubation (Table 2). We observed a time-dependent loss of acidification that correlates with lysosomal impairment. As aforementioned, drug-mediated induction of lysosome alkalization has been related to a blockage of the autophagic flux and high rates of apoptosis [33,34], and mitochondrial dysfunction is considered to be one of the main causes of the impairment of lysosomes [35]. With this in mind, changes in mitochondrial membrane potential (ψ_m_) were analyzed after 24 h and 48 h incubation with the drug combination (Table 2). We found a time-dependent increase in cell population with an altered value of ψ_m_, which suggests a disruption on normal mitochondrial function. Since a significant loss of ψ_m_ was noticed after 24 h, prior to lysosomal alkalization (Table 2), mitochondrial dysfunction seems to be an upstream event relative to lysosomal impairment. 

Finally, we measured reactive oxygen species (ROS) levels due to their key role in mitochondrial homeostasis. A significant increase in ROS production was observed after a short incubation time (1 h) and it was maintained upon overnight treatment with the drug combination (Table 3). The role of ROS on cell death was further confirmed by pre-incubation of Caco-2 cells with the ROS scavenger N-acetylcysteine (NAC) (3 mM, 1 h), which reduced the great decrease in cell viability caused by 72 h treatment with the drug combination (Table 3). Therefore, our data suggest that disruption of redox balance and mitochondrial dysfunction with lysosomal impairment and autophagy blockage triggered by drug combination, results in apoptotic cell death. 

## 4. Conclusions

We have investigated the anticancer potential of a novel drug combination made from the acidic polyphenols extracted from rosehips of the wild plant *Rosa canina* and the gold complex (Au(C≡C-2-NC_5_H_4_)(PTA)). Assays performed on the human colorectal adenocarcinoma Caco-2 cell line showed that the addition of increasing concentrations of acidic polyphenols significantly enhanced the antiproliferative effect of the gold derivative, and reduced the drug exposure time necessary to obtain a significant decrease in viability of cancer cells. The resulting drug combination triggered strong apoptosis that might be a consequence of a blockage of the autophagic flux. Regarding the unpaired autophagy, the marked pro-oxidant effect of the drug combination led to a disruption of mitochondrial function, which might be responsible for the lysosome alkalinization observed. To our knowledge, this is the first in-vitro study that analyzes the potential antitumor effect of a combination of a plant-derived extract and a gold complex. Taken together, our results suggest a potential role of plant polyphenols as adjuvant for gold-based drugs on colorectal cancer therapy. Future studies will be necessary to determine the possible interaction between polyphenols and ligands of the gold complex tested.

## Figures and Tables

**Table 1:** Cell viability. Measurement of cell viability of Caco-2 cells upon 24 h treatment with DC in presence or not of necrostatin-1 (50 μM, 1 h pre-incubation).

% Cell viability	
Control	100 ± 0.00
DC	69.27 ± 0.64*
DC+Nec-1	72.14 ± 6.61*

No significant changes were observed between DC with/without Nec-1. * *p* < 0.05 vs control.

**Table 2:** Analysis of lysosomal and mitochondrial integrity. Above: percentages of Caco-2 cells with functional (acid) lysosomes upon 24 h and 48 h incubation with the drug combination (DC). Below: changes in mitochondrial membrane potential (ψ_m_) of Caco-2 cells after 24 h and 48 h incubation with DC.

**% Cells with Functional (Acid) Lysosomes**		
	**24 h**	**48 h**
Negative control	85.50 ± 3.11	94.10 ± 2.26
DC	85.55 ± 1.62	74.95 ± 1.62*
	**% Cells with Conserved ψ_m_**	
	**24 h**	**48 h**
Negative control	100.00 ± 0.00	100.00 ± 0.00
DC	61.98 ± 10.68*	27.25 ± 8.52*

* *p* < 0.05 vs negative control.

**Table 3:** Role of reactive oxygen species (ROS) levels on cell death triggered by DC. Above: measurement of ROS levels expressed as arbitrary units of fluorescence in Caco-2 cells incubated for 1 h and 24 h with the drug combination (DC). Fluorescence data were normalized with cell viability. Below: analysis of Caco-2 cell viability pre-incubated with NAC and then treated 24 h and 72 h with DC.

	**ROS levels (Arbitrary Units)**	
	**1 h**	**24 h**
Negative control	1 ± 0.00	1 ± 0.00
DC	1.32 ± 0.10*	1.31 ± 0.04*
	**% Cell Viability**	
	**24 h**	**72 h**
Negative control	100 ± 0.00	100 ± 0.00
DC	68.13 ± 7.73*	27.94 ± 1.50*
DC + NAC	72.42 ± 0.47*	80.16 ± 2.52*#

* *p* < 0.05 vs negative control. # *p* < 0.05 vs treatment with DC.

## Supplementary Materials

The following are available online at https://www.mdpi.com/2076-3921/9/1/17/s1, Figure S1. Optical phase contrast microscopy at 20X magnification of Caco-2 cells. Above: differentiated cells at one week post-seeding, (**a**) definition of cytoplasmic membrane limits and the polygonal shape. Below: undifferentiated cells at 24 h post-seeding, (**b**) undefined cytoplasmic membrane and round shape. The low density seed cells shows a bigger size than the differenced cells. Figure S2: title: Measurement of differentiated Caco-2 cells viability 72 h after incubation with drug combination (DC). Figure S3. Title: Cell death analysis of Caco-2 cells viability 48 h after incubation. Figure S4. Title: Flow cytometry analysis of the expression levels of RIP-1 on Caco-2 cells 48 h after incubation. Figure S5. Title: Measurement of changes in the formation of autophagosomes in Caco-2 cells at 24 h post-incubation.

## Abbreviations

PTA: 1:3:5-triaza-7-phosphadamantane, AP: acidic polyphenols, CRC: colorectal cancer, CQ: chloroquine, DCF: dichlorofluorescein, DMEM: Dulbecco’s Modified Eagles Medium, DMSO: dimethyl sulfoxide, DC: drug combination, GC: gold complex, ψ_m_: mitochondrial membrane potential, MTT: 3-(4,5-dimethyl-2-thiazoyl)-2,5-diphenyltetrazolium bromide, NAC: N-acetylcysteine, Nec-1: necrostatin-1, PBS: phosphate buffered saline, ROS: reactive oxygen species, RIP-1: receptor-interacting protein kinase 1, SRB: sulforhodamine B, TE: total extract.
