# Supplementary material for: A Combination of Rosa Canina Extracts and Gold Complex Favors Apoptosis of Caco-2 Cells by Increasing Oxidative Stress and Mitochondrial Dysfunction
Source: Antioxidants (Basel). 2019 Dec 24;9(1):17. doi: 10.3390/antiox9010017 (PMC7023183; doi:10.3390/antiox9010017)
Supplement: Supplementary file 1 [file antioxidants-09-00017-s001.pdf]

A combination of *Rosa canina* extracts and gold complex favors apoptosis of Caco-2 cells by increasing oxidative stress and mitochondrial dysfunction

Inés Mármol[1], Nerea Jiménez-Moreno[2], Carmen Ancín-Azpilicueta[2], Jesús Osada[3, 4], Elena Cerrada[5*], Maria Jesús Rodríguez-Yoldi[1, 4*]

[1]Dept. of Pharmacology and Physiology, Veterinary Faculty, University of Zaragoza, Zaragoza, Spain.

[2]Dept. of Science, Public University of Navarra, INAMAT, Navarra, Spain.

[3]Dept. of Biochemistry and Molecular Biology, Veterinary Faculty, University of Zaragoza, Zaragoza, Spain.

[4]CIBERobn (ISCIII), IIS Aragón, IA2, Zaragoza, Spain.

[5]Dept. of Inorganic Chemistry, Sciences Faculty, University of Zaragoza, Zaragoza, Spain.

*Corresponding authors: M. J. Rodriguez-Yoldi,

Physiology, Veterinary Faculty,

Miguel Servet 177, 50013 Zaragoza, Spain

Phone : 34-976 761649. Fax : 34-976 761612

e-mail: mjrodyol@unizar.es.

*Corresponding authors: E. Cerrada,

Inorganic Chemistry, Instituto de Síntesis Química y Catálisis Homogénea-ISQCH,

Pedro Cerbuna 12, 50009 Zaragoza, Spain

Phone : 34-876 553529

e-mail: ecerrada@unizar.es

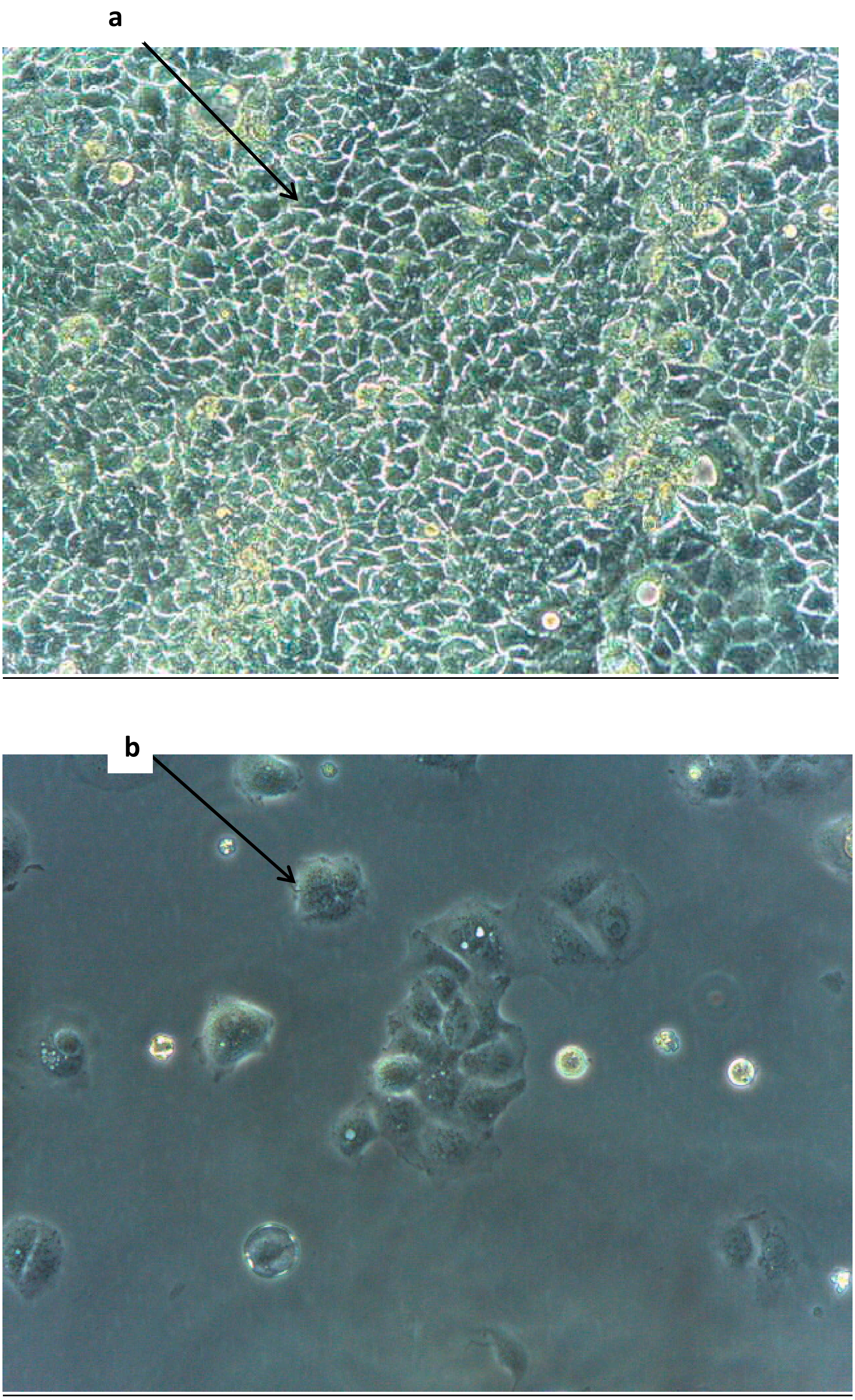

Figure S1. Optical phase contrast microscopy at 20X magnification of Caco-2 cells. Up) Differentiated cells at one week post-seeding, (**a**) definition of cytoplasmic membrane limits and the polygonal shape Down) Undifferentiated cells at 24 h post-seeding., (**b**)

undefined cytoplasmic membrane and round shape. The low density seed cells shows a bigger size than the differenced cells

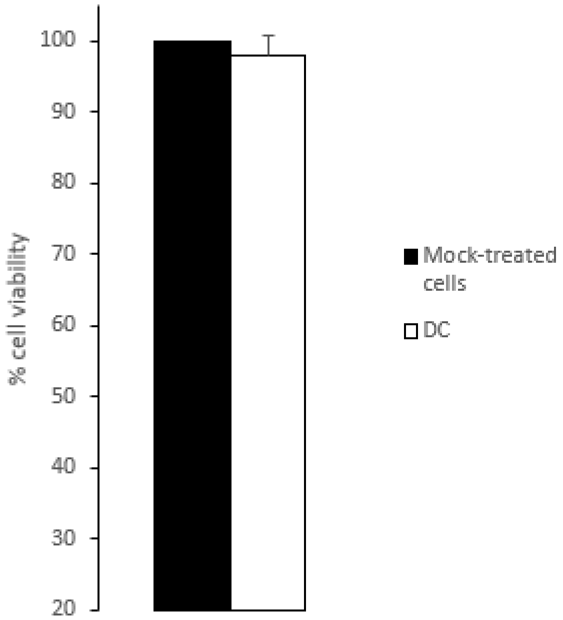

Figure S2. Measurement of differentiated Caco-2 cells viability after 72 h incubation with drug combination (DC). No significant changes were observed in comparison to negative control.

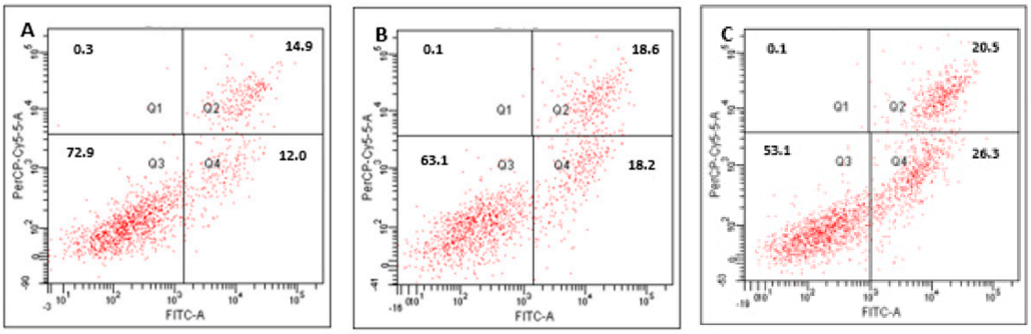

Figure S3. Cell death analysis of Caco-2 cells viability after 48h incubation with A) DMSO (negative control), **B**) gold complex and **C**) acidic polyphenols. Q1: necrotic cells; Q2: late apoptotic cells; Q3: alive cells; Q4: early apoptotic cells. Percentages of cell population on each condition are included.

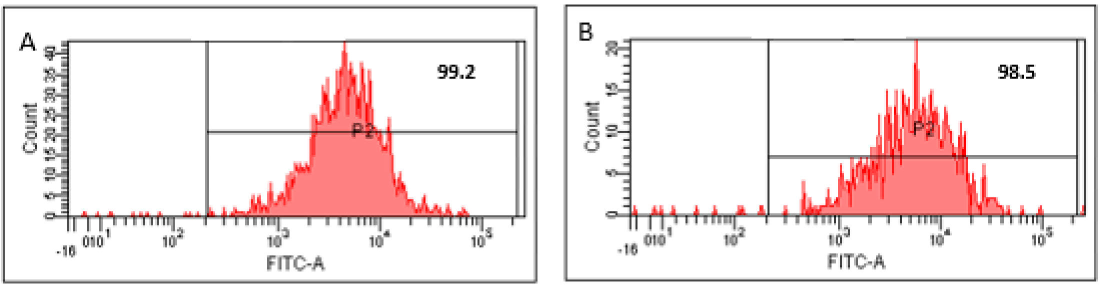

Figure S4. Flow cytometry analysis of the expression levels of RIP-1 on Caco-2 cells after 48h incubation with **A**) DMSO (negative control) or **B**) Drug combination (DC). Percentages of cell population with non-active RIP-1 (P2) are included.

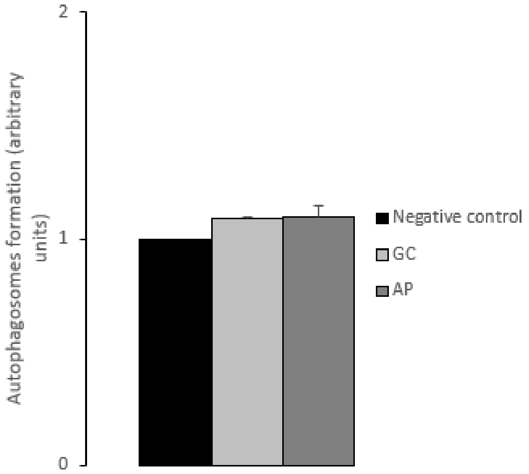

Figure S5. Measurement of changes in the formation of autophagosomes on Caco-2 cells after 24 h incubation with the gold complex (GC) and acidic polyphenols (AP). No significant changes were observed in comparison to negative control.
